# Characterising complex health needs and the use of preventive therapies in the older population: a population-based cohort analysis of UK primary care and hospital linked data

**DOI:** 10.1186/s12877-023-03770-z

**Published:** 2023-01-31

**Authors:** Leena Elhussein, Annika M. Jödicke, Ying He, Antonella Delmestri, Danielle E. Robinson, Victoria Y. Strauss, Daniel Prieto-Alhambra

**Affiliations:** Pharmaco- and Device Epidemiology, Centre for Statistics in Medicine, Nuffield Department of Orthopaedics, Rheumatology and Musculoskeletal Sciences (NDORMS), University of Oxford, Windmill Road, Oxford, United Kingdom

**Keywords:** Geriatric medicine, Older people, Preventive therapies, Pharmaco-epidemiology, Frailty, Polypharmacy

## Abstract

**Background:**

While several definitions exist for multimorbidity, frailty or polypharmacy, it is yet unclear to what extent single healthcare markers capture the complexity of health-related needs in older people in the community. We aimed to identify and characterise older people with complex health needs based on healthcare resource use (unplanned hospitalisations or polypharmacy) or frailty using large population-based linked records.

**Methods:**

In this cohort study, data was extracted from UK primary care records (CPRD GOLD), with linked Hospital Episode Statistics inpatient data. People aged > 65 on 1st January 2010, registered in CPRD for ≥ 1 year were included. We identified complex health needs as the top quintile of unplanned hospitalisations, number of prescribed medicines, and electronic frailty index. We characterised all three cohorts, and quantified point-prevalence and incidence rates of preventive medicines use.

**Results:**

Overall, 90,597, 110,225 and 116,076 individuals were included in the hospitalisation, frailty, and polypharmacy cohorts respectively; 28,259 (5.9%) were in all three cohorts, while 277,332 (58.3%) were not in any (background population). Frailty and polypharmacy cohorts had the highest bi-directional overlap. Most comorbidities such as diabetes and chronic kidney disease were more common in the frailty and polypharmacy cohorts compared to the hospitalisation cohort. Generally, prevalence of preventive medicines use was highest in the polypharmacy cohort compared to the other two cohorts: For instance, one-year point-prevalence of statins was 64.2% in the polypharmacy cohort vs. 60.5% in the frailty cohort.

**Conclusions:**

Three distinct groups of older people with complex health needs were identified. Compared to the hospitalisation cohort, frailty and polypharmacy cohorts had more comorbidities and higher preventive therapies use. Research is needed into the benefit-risk of different definitions of complex health needs and use of preventive therapies in the older population.

**Supplementary Information:**

The online version contains supplementary material available at 10.1186/s12877-023-03770-z.

## Introduction

With society growing older, the number of people suffering from multimorbidity, frailty and polypharmacy is rapidly increasing in the UK [1, 2]. These people often require complex clinical management, have higher healthcare utilisation, and are more prone to adverse events and mortality [1–3]. Whilst several definitions exist for multimorbidity, polypharmacy, and frailty, it is yet unclear to what extent single healthcare markers capture the complexity of health-related needs in older people in the community.

As part of its strategy to optimise care for multimorbid patients, the UK’s National Institute for Health and Care Excellence (NICE) requested research into identifying patients with complex health needs based on primary care electronic health records [3]. In particular, their multimorbidity guideline suggested to consider validated tools such as the electronic frailty index (eFI) [4] to identify multimorbid patients at risk for adverse events and unplanned hospital admissions, as well as markers of polypharmacy to identify patients with high treatment burden.

The objective of this study was to identify and characterise older patients with complex health needs, aiming to enhance further research into this important patient group. We selected patients based on three distinct health markers (unplanned hospitalisation, frailty and polypharmacy), compared their characteristics to the background population, and explored the overlap between the different cohorts. Subsequently, we assessed the utilisation of common preventive drugs in people with complex health needs, as the evidence behind current recommendations for their use in multimorbid patients with limited life expectancy is scarce.

## Materials and methods

### Data source

We extracted data from the UK Clinical Practice Research Datalink (CPRD) GOLD, a primary care database containing > 2.8million anonymised records of active patients (September 2019) [5]. CPRD was representative of the UK population in terms of age, sex and ethnicity [6]. We linked CPRD data to Office for National statistics (ONS) mortality data, a UK death registry, and to two Hospital Episodes statistics datasets (HES Admitted Patient Care, and Accident and Emergency), which contain data on hospital admissions and emergency room attendance. These HES datasets will be referred to as CPRD-HES.

### Population

All subjects with linkages to CPRD-HES at study start (01/01/2010), aged > 65 years, acceptable for clinical research, who were registered with an up-to-standard (UTS) practice for at least 1 year comprised the source population. In CPRD GOLD, each practice is associated to an UTS date, which represents the starting point when data in the practice are considered to have continuous high-quality and therefore are suitable for research purposes. Moreover, in CPRD GOLD each patient is labelled as ‘acceptable’ for use in research by a checking process that identifies individuals with continuous follow up and with valid clinical records [6].

### Cohort definitions

Among the source population, we identified three cohorts of older patients with ‘complex health needs’ based on the following health-care markers:

- a) Hospitalisation cohort: defined by the number of unplanned hospital admissions (HES Admitted Patient Care, and Accident and Emergency), identified in the linked HES data in 2009.
- b) Frailty cohort: defined using the validated eFI score developed by Clegg et al. [4], and based on the count of frailty markers/deficits as recorded during 2009.
- c) Polypharmacy cohort: defined by the number of different drug substances prescribed in 2009.

After examining the data distribution of these markers, patients in the top quintile (20%) constituted the respective cohorts. If there was no clear cut-off to determine the top quintile due to count data, the nearest division based on the data distribution, or a pre-existing clinical cut-off was chosen. Patients could be included to multiple cohorts at the same time. Patients who belonged to all three cohorts constituted the overlap group, whereas those who did not belong to any of the three cohorts comprised the background population.

### Utilisation of preventive therapies

The NICE multimorbidity guideline recommended research into continuing and stopping of three preventive therapies, namely bisphosphonates, statins, and anti-hypertensives, as those may potentially be avoidable in patients with limited life expectancy and frailty [4]. We evaluated the use of oral bisphosphonates, statins, and anti-hypertensives, separately in each cohort. Prescriptions of preventive treatments were identified based on product codes in CPRD (Code lists available in Additional File 1). Anti-hypertensives were analysed as a group, and separately by drug class according to the WHO-ATC classification. We calculated treatment episodes by combining individual prescriptions, allowing for a maximum 90-day gap between prescriptions and with adding a 90-day washout period at the end of each episode.

### Statistical analyses

We calculated one month, three month, and one year point prevalence (PP) prior to start date to identify prevalent users of oral bisphosphonates, statins, and anti-hypertensives in each cohort, the overlap group, and background population, separately. Furthermore, we calculated one-year treatment incidence rates (IR) in the first year after start date after excluding one-year prevalent patients of each drug class for the same groups. We assumed a Poisson distribution for both analyses. Incidences were counted as the first prescription after start date and IRs were calculated per 1000 person years. Patients exited the study at the earliest of practice last collection date, patient transfer-out of practice date, or death date [7]. For IR analysis, a patient would contribute to the sum of person-years until a prescription of the preventive therapy is reported. We used STATA (Version 15.1) for cohort identification, and R (Version 3.6.0) for cohort characterisation and calculation of PPs and IRs.

## Results

### Cohort identification

Cut-offs for inclusion to each of the three cohorts were: ≥ 1 unplanned hospital admission for the hospitalisation cohort, an eFI score ≥ 3 for the frailty cohort, and ≥ 10 medicines for the polypharmacy cohort. Figure S1 and Table S1 in Additional File 2 describe the distribution of these markers.

**Fig. 1:**
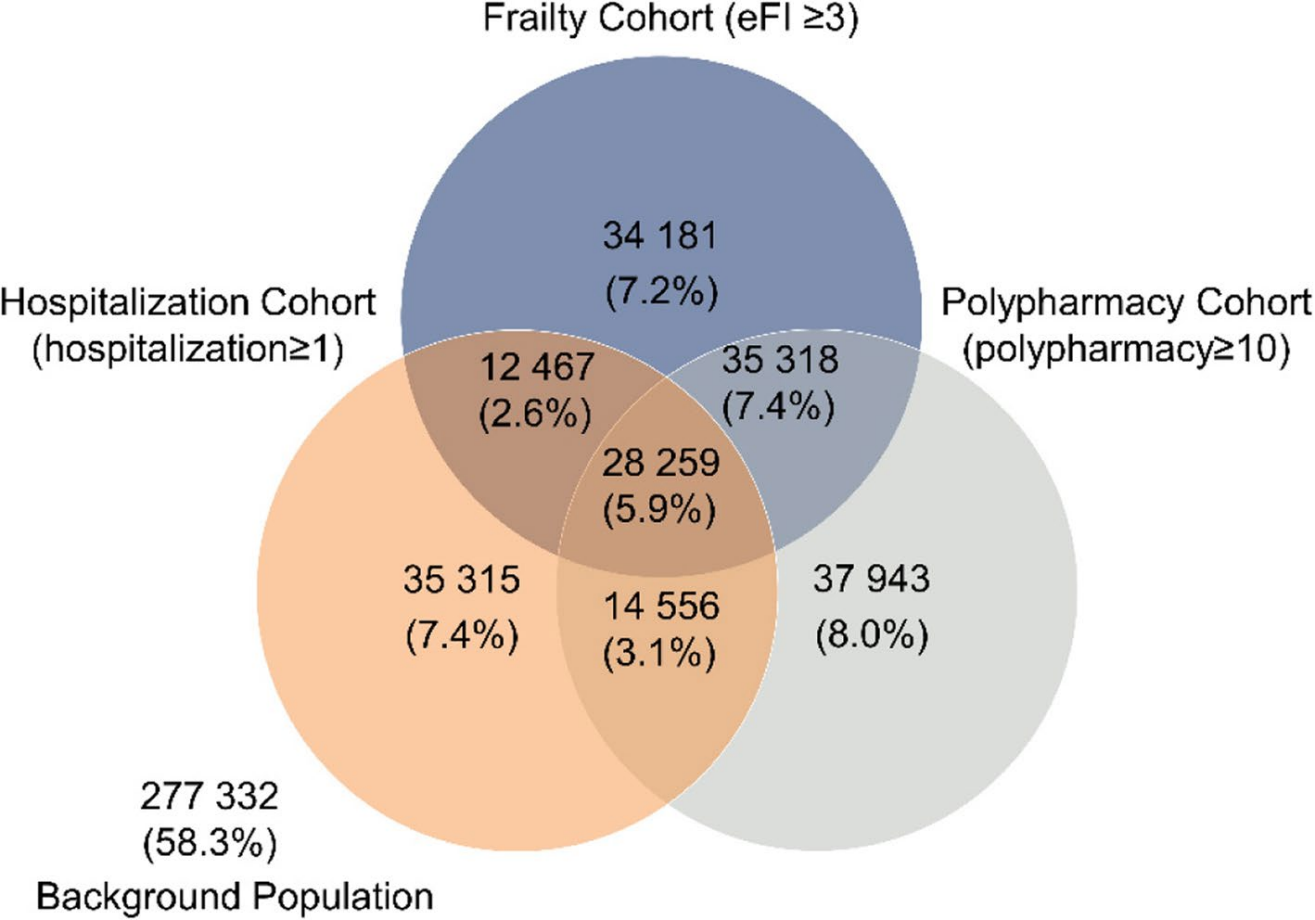
Venn diagram: Number of patients in each cohort of complex health needs, and cohort overlap

Figure 1 shows the distribution of the three cohorts. Of the 475 371 eligible people, 198 039 (41.3%) belonged to at least one cohort, while 277 332 (58.3%) did not belong to any cohort and constituted the *background* population. Frailty and polypharmacy had the highest bi-directional overlap (> 50%).

### Cohort characteristics

Mean age was similar in the three cohorts and the overlap group, (between 78.2 years and 79.7 years), and slightly lower in the background population (74.7 years). The proportion of males was lower in the three cohorts and the overlap group (between 39.3% and 43.3%) compared to the background population, (46.7%). Table 1 describes the baseline characteristics of the three complex health needs cohorts, the overlap group and the background population.

**Table 1:** Baseline characteristics

	**Hospitalisation Cohort**	**Frailty Cohort**	**Polypharmacy Cohort**	**Overlap group**	**Background population**
n	90,597	110,225	116,076	28,259	277,332
Hospitalisation Cohort	90,597 (100.0%)	40,726 (36.9%)	42,815 (36.9%)	28,259 (100.0%)	0 (0)
Frailty Cohort	40,726 (45.0%)	110,225 (100.0%)	63,577 (54.8%)	28,259 (100.0%)	0 (0)
Polypharmacy Cohort	42,815 (47.3%)	63,577 (57.7%)	116,076 (100.0%)	28,259 (100.0%)	0 (0)
Gender = Male	39,201 (43.3%)	45,272 (41.1%)	45,578 (39.3%)	11,470 (40.6%)	129,406 (46.7%)
Age (mean (SD))	78.16 (7.76)	78.74 (7.41)	78.19 (7.38)	79.66 (7.41)	74.73 (6.97)
Socio-economic status^a^					
1 (least deprived)	20,559 (22.7%)	24,226 (22.0%)	25,520 (22.0%)	5632 (19.9%)	78,067 (28.1%)
2	21,641 (23.9%)	25,823 (23.4%)	26,496 (22.8%)	6340 (22.4%)	72,208 (26.0%)
3	19,291 (21.3%)	23,633 (21.4%)	24,482 (21.1%)	6003 (21.2%)	57,973 (20.9%)
4	16,921 (18.7%)	21,331 (19.4%)	22,730 (19.6%)	5760 (20.4%)	44,379 (16.0%)
5 (most deprived)	12,112 (13.4%)	15,134 (13.7%)	16,744 (14.4%)	4501 (15.9%)	24,525 (8.8%)
BMI (mean (SD))	27.10 (5.55)	27.95 (5.78)	28.31 (5.86)	27.59 (5.97)	26.73 (4.66)
Charlson Comorbidity Index^b^ (mean (SD))	0.37 (0.92)	0.51 (1.02)	0.38 (0.91)	0.64 (1.15)	0.08 (0.42)
General Practitioner visits^b^ (median IQR])	12 [7,19]	14 [9,21]	15 [10,22]	19 [13,27]	5 [2,8]
eFI^b^ (median [IQR])	2 [1,3]	3 [3,4]	3 [2,4]	4 [3,5]	1 [0, 1]
Unplanned hospital admissions^b^ (median [IQR])	1 [1,2]	0 [0, 1]	0 [0, 1]	1 [1,2]	0 [0, 0]
Number of different drugs^b^ (median [IQR])	9 [6,13]	10 [8,14]	13 [11,15]	14 [12,18]	4 [1,6]

*BMI* Body mass index, e*FI* electronic Frailty Index

^a^ Socio-economic status determined by an Index of Multiple Deprivation (IMD2004_5) made at small area level and grouped into quintiles

^b^ Calculated based on the year prior to start

Missingness was around 0.1% for socio-economic status, and ranged between 19.8% to 64.1% for BMI

### Comorbidities

**Fig. 2:**
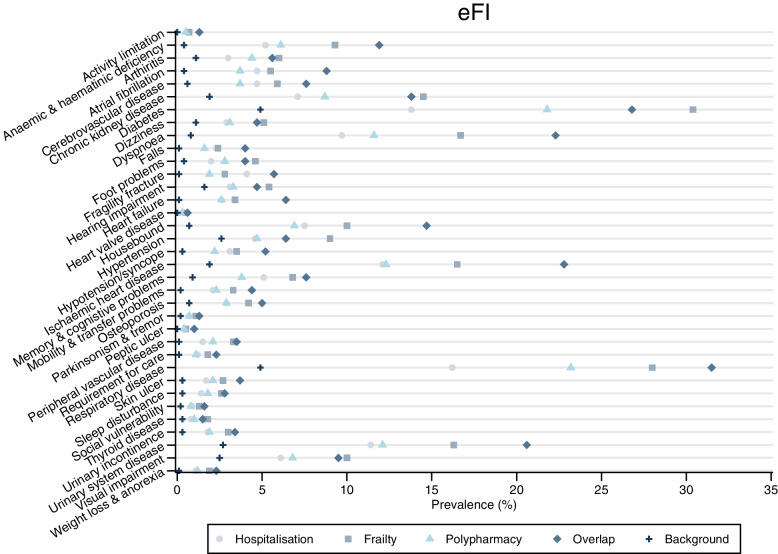
Prevalence of eFI deficits/conditions—in the year prior to start—for all cohorts, overlap and background population

Comorbidity burden was higher in the three complex health needs cohorts and the overlap group compared to the background population, especially for diabetes, ischemic heart disease, respiratory disease, and diseases of the urinary system. For most conditions, frailty cohort had the highest prevalence, followed by the polypharmacy cohort. The hospitalisation cohort had the highest prevalence of myocardial infarction, cancer, and fragility fractures. Figure 2 and Figure S2 in Additional File 2 show the prevalence of each individual condition included in the eFI score and Charlson Comorbidity Index in the year prior to start date, respectively.

### Preventive therapy use

#### Point prevalence

**Fig. 3:**
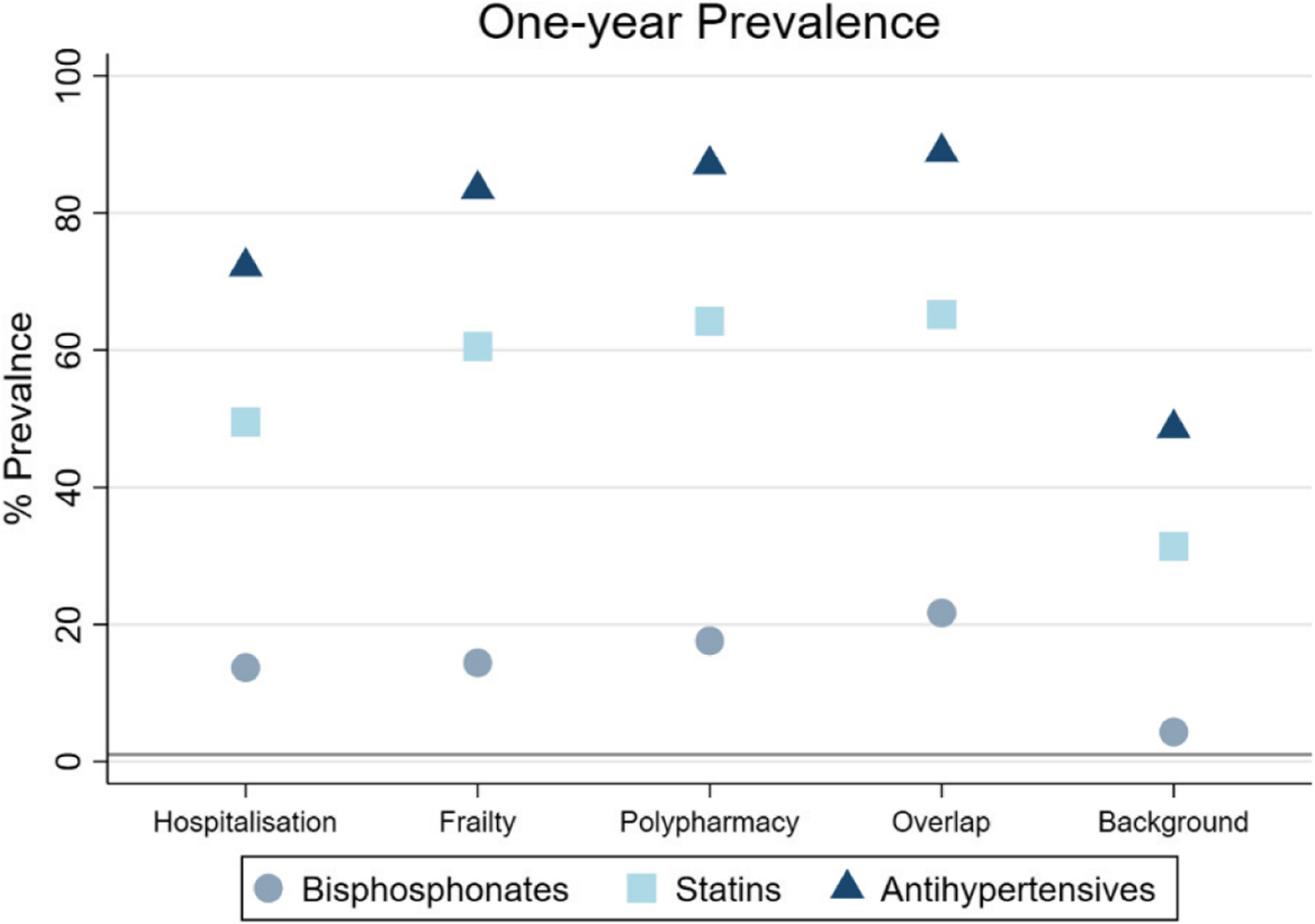
One-year Point Prevalence for oral bisphosphonates, statins and anti-hypertensives, for all cohorts

Figure 3 describes one-year PP of oral bisphosphonates, statins, and anti-hypertensives for all cohorts. Compared to the hospitalisation and frailty cohort, the polypharmacy cohort had a higher prevalence of preventive treatment use. Prevalence was highest in the overlap group and lowest in the background population. Among the three preventive therapies, the use of anti-hypertensives was substantially higher than for oral bisphosphonates and statins for all cohorts (≥ 70.0%). Figure S3 in Additional File 2 describes one and three-month PP, and Tables S2a-e in Additional File 2 report PP of the different anti-hypertensive classes.

#### Incidence

Table 2 shows one-year IRs of oral bisphosphonates, statins, and anti-hypertensives for all cohorts. Both the frailty and polypharmacy cohort had similar IRs for the preventive therapies, with hospitalisation cohort having the lowest IRs among the three cohorts in most cases. As with point prevalence, the overlap group had generally higher IRs than the three identified cohorts. Tables S3a-e in Additional File 2 describe the one-year IRs for the different classes of anti-hypertensives.

**Table 2:** One-year IRs – excluding patients with one-year prevalence

	**Bisphosphonates**		
	**N**	**PY**	**IR (95% CI)**
**Hospitalisation cohort**	2233	69,984.6	31.91 (30.6, 33.2)
**Frailty cohort**	2441	85,996.6	28.39 (27.3, 29.5)
**Polypharmacy cohort**	2477	87269.8	28.38 (27.3, 29.5)
**Overlap group**	780	19,081.4	40.88 (38.0, 43.8)
**Background population**	2604	253,071.6	10.29 (9.9, 10.7)
	**Statins**		
	**N**	**PY**	**IR (95% CI)**
**Hospitalisation cohort**	2472	39,902.3	61.95 (59.5, 64.4)
**Frailty cohort**	2520	38,216.8	65.94 (63.4, 68.5)
**Polypharmacy cohort**	2327	36515.6	63.73 (61.1, 66.3)
**Overlap group**	551	8115.8	67.89 (62.2, 73.6)
**Background population**	8425	177,430.1	47.48 (46.5, 48.5)
	**Anti-hypertensives**		
	**N**	**PY**	**IR (95% CI)**
**Hospitalisation cohort**	2793	21,610.1	129.25 (124.5, 134.0)
**Frailty cohort**	2400	15,449.0	155.35 (149.1, 161.6)
**Polypharmacy cohort**	1972	12759.6	154.55 (147.7, 161.4)
**Overlap group**	491	2481.901	197.83 (180.3, 215.3)
**Background population**	9622	131,709.3	73.06 (71.6, 74.5)

*N* Number of incident users, *PY* person years, *IR* incidence rate

## Discussion

### Principal findings

We identified cohorts of older patients with complex healthcare needs, based on healthcare resource use (unplanned hospitalisations, polypharmacy) or frailty. Compared to the background population, patients included in either of the cohorts were older, had a higher comorbidity burden, higher number of prescribed drugs, and a substantially higher prevalence and incidence of preventive drug use.

### Assessment of complex healthcare needs in the older population

#### Identification of patients with complex health needs

Following the recommendation of the NICE multimorbidity guideline [3], we used UK primary care data and inpatient records to select patients with complex health needs. The utilisation of primary care data allows for a systematic and comprehensive approach to identify potential target groups for preventive measures, who might benefit most from care optimisation. We defined patients with “complex health needs” as those individuals belonging to the top quintile of the distribution of the respective heath markers. While multiple definitions for polypharmacy and frailty are used side-by-side, we opted for a data-driven approach to identify the most vulnerable among the older population, allowing for a uniform definition applicable in different healthcare settings, data types and patient collectives. Access to large and representative population-based data allowed us to anchor our definitions based on their public health relevance.

Although the association between frailty, polypharmacy and healthcare utilisation has been described previously [4, 8–15], no direct comparison of patient groups identified by these distinct markers has been done.

##### Polypharmacy

While no standard is agreed upon [16–18], the concomitant prescription of ≥ 5 medicines is considered polypharmacy, with ≥ 10 prescriptions referred to as excessive, severe or hyper-polypharmacy [2, 18]. While our approach to cumulatively assess drug use might overestimate the number of drugs taken concomitantly, it reflects the patients’ treatment burden with respect to both acute and chronic indications over a long time, providing a pragmatic screening tool.

##### Healthcare utilisation

Prevention of unplanned and avoidable hospitalisations are a public health priority, as they present a major burden for the affected patients and substantial costs for the health care system. Our study found that 19.1% of the study population was admitted to hospital. Visits with general practitioners were significantly more common in the frailty, polypharmacy and overlap cohort compared to the background population, highlighting their complex health needs.

##### Frailty

While drug prescriptions and unplanned hospitalisations are well recorded in real world data, the assessment of a patients' frailty status is challenging. Physical and mental fitness vary greatly among patients of the same age and while a patient's frailty will greatly influence a general practitioners treatment decision, such characteristics are typically not recorded in primary care databases. Therefore, frailty is one of the most important confounders in pharmaco-epidemiological studies among the older population.

The concept of frailty rapidly evolved over the last two decades, with the two major concepts of “frailty phenotype” and “frailty indexes” being established [8]. In recent years, different approaches have been developed to identify frailty in routinely collected data [4, 19–22].

#### Association between polypharmacy, frailty and unplanned hospitalisation

To our knowledge, this is the first study directly comparing three different markers of complex heath needs to characterise patient groups. The overlap of the cohorts identified based on single health markers illustrate the association between polypharmacy, frailty and hospitalisation. A recent systematic review summarised the bi-directional association between frailty and polypharmacy [8]: mean drug consumption was reported to be higher among frail older patients compared to robust individuals, and increased likelihood for frailty was described among patients with polypharmacy (OR 1.77–2.55) [11–13] and hyper-polypharmacy (OR 4.47–5.8) [11–13], respectively. In addition, frailty among the UK’s community-dwelling older was associated with reduced quality of life [14], increased annual GP consultations, hospital admissions and elevated healthcare costs [4, 23].

Our results are in line with the associations described in the literature, with a large pairwise overlap between hospitalisation, frailty and polypharmacy cohort. However, we also found a substantial number of patients only included in *one* of the respective cohorts. This highlights that, while there is a strong association and correlations exist between them, different markers of complex heath needs are distinct and complementary. Future studies should refrain from considering single healthcare markers as interchangeable when selecting a geriatric study population or adjusting for comorbidity, frailty, or complexity. Instead, future research should rather take into account all and each available markers of complexity at a time as they might complement each other. For those studies where only a single healthcare marker is of interest to define a population, clear definitions are essential to facilitate comparison of study populations and findings across different healthcare settings, countries, and time periods.

### Cohort characteristics and use of preventive treatments in the older people

#### Comorbidity burden

Multimorbidity is often poorly reported and definitions vary greatly between individual studies [24]. Consistent across different measures, comorbidity burden in our complex health needs cohorts was substantially higher than in the background population. As counts of deficits were used to define the frailty cohort, it was to be expected that the highest prevalence of comorbidities would be found in the frailty cohort and overlap group.

#### Preventive treatments

Patients with complex healthcare needs were commonly prescribed preventive treatments, with both incidence and prevalence being significantly higher than for the background population. Direct comparison of use of preventive therapies to previous observational studies is difficult considering differences in patient collectives, age-group definitions, and treatment indication [25, 26] between individual studies. However, in the UK, annual initiation rates of statins previously reported for people aged 60–84 years were between 30–50/1000 person-years [27], which is largely comparable to our results. Likewise, incidence rates for anti-osteoporosis treatment (12.5 to 26.0/1000 person-years) were previously reported for women aged 65–84 years [28], with considerably lower rates reported in males. UK based studies reporting incidence rates of antihypertensive treatment among the older population are scarce. A CPRD based study reports annual IR of approximately 15% in 2001 assessing hypertensive patients aged ≥ 40 years with ≥ 3 cardiovascular risk factors [29]. In our study, incidence rates varied across the different cohorts, with IR 73.1/1000 and 197.8/1000 patient years for background population and overlap group.

### Strengths and limitations

Our study comes with both strengths and limitations. We consider the use of CPRD, which is frequently used for pharmaco-epidemiological studies [30] and provides good quality records for research, a particular strength. In addition, CPRD is representative for the UK population, which enables the use of results from our study to inform local public health strategies for older residents. Another strength of this study is the direct comparison of the different definitions of complex healthcare needs and their impact on which patients are included into the cohort.

We defined our frailty cohort based on the eFI score. When describing the association between frailty and polypharmacy, it is important to consider that polypharmacy (defined as ≥ 5 medicines per year) is included as a deficit in the eFI score. Thus, there is an operational association, which may overestimate the true overlap between cohorts. While primary care records include all prescriptions issued by GPs, drugs purchased over-the-counter and prescribed in hospital are not included. Therefore, the true burden of polypharmacy may be underestimated. In addition, no information on the appropriateness of polypharmacy in the individual patients was available. While future studies should use validated criteria to assess potentially inappropriate medication in older people with complex health needs, this was beyond the scope of the present work. Lastly, for practical reasons, we assessed all patient information using a baseline period of one year. Therefore, underreporting of chronic deficits cannot be ruled out.

## Conclusion

Our study shows that depending on the definition used, cohorts of patients with complex healthcare needs slightly differ with respect to their demographic characteristics, comorbidity pattern and drug utilisation. Our results highlight that several indicators of complex healthcare needs should be used in any future research among geriatric populations, as they may complement each other. We found the use of preventive therapies to be common among older patients with complex healthcare needs. Future studies should therefore evaluate the risk–benefit of these medications in older patients with reduced lifespan and high potential for adverse drug reactions [31].

## Supplementary Information

**Additional file 1.** Code lists.

**Additional file 2: Table S1.** descriptive statistics of variables used to identify cohorts. **Figure S1.** Histograms of variables used to identify cohorts of complex health needs: (**a**) unplanned hospital admission, (**b**) eFI and (**c**) polypharmacy. **Figure S2.** Prevalence of Charlson morbidity index deficits/conditions - in the year prior to start - for all cohorts, overlap and background population. **Table S2.** **a** Point prevalence of the different antihypertensives classes for the Hospitalisation cohort. **b**: Point prevalence of the different antihypertensives classes for the frailty cohort. **c**: Point prevalence of the different antihypertensives classes for the polypharmacy cohort. **d**: Point prevalence of the different antihypertensives classes for the overlap group. **e**: Point prevalence of the different antihypertensives classes for the background population. **Table S3.** **a**: One-year IRs – excluding one-year prevalence of the different antihypertensives classes for the Hospitalisation cohort. **b**: One-year IRs – excluding one-year prevalence of the different antihypertensives classes for the frailty cohort. **c**: One-year IRs – excluding one-year prevalence of the different antihypertensives classes for the polypharmacy cohort. **d**: One-year IRs – excluding one-year prevalence of the different antihypertensives classes for the overlap group. **e**: One-year IRs – excluding one-year prevalence of the different antihypertensives classes for the background population.

## Data Availability

The data that support the findings of this study are available from CPRD (https://cprd.com/) subject to protocol approval. Anonymised data were accessed under the Oxford University CPRD multi-study license for the current study. The datasets analysed during the current study are not publicly available due to data governance reasons, but code to process the study dataset from CPRD data is available from the corresponding author [annika.jodicke@ndorms.ox.ac.uk] on reasonable request.
