# Supplementary material for: Characterising complex health needs and the use of preventive therapies in the older population: a population-based cohort analysis of UK primary care and hospital linked data
Source: BMC Geriatr. 2023 Jan 31;23:58. doi: 10.1186/s12877-023-03770-z (PMC9890735; doi:10.1186/s12877-023-03770-z)
Supplement: Supplementary file 2 — Additional file 2: Table S1. descriptive statistics of variables used to identify cohorts. Figure S1. Histograms of variables used to identify cohorts of complex health needs: (a) unplanned hospital admission, (b) eFI and (c) polypharmacy. Figure S2. Prevalence of Charlson morbidity index deficits/conditions - in the year prior to start - for all cohorts, overlap and background population. Table S2. a Point prevalence of the different antihypertensives classes for the Hospitalisation cohort. b: Point prevalence of the different antihypertensives classes for the frailty cohort. c: Point prevalence of the different antihypertensives classes for the polypharmacy cohort. d: Point prevalence of the different antihypertensives classes for the overlap group. e: Point prevalence of the different antihypertensives classes for the background population. Table S3. a: One-year IRs – excluding one-year prevalence of the different antihypertensives classes for the Hospitalisation cohort. b: One-year IRs – excluding one-year prevalence of the different antihypertensives classes for the frailty cohort. c: One-year IRs – excluding one-year prevalence of the different antihypertensives classes for the polypharmacy cohort. d: One-year IRs – excluding one-year prevalence of the different antihypertensives classes for the overlap group. e: One-year IRs – excluding one-year prevalence of the different antihypertensives classes for the background population. [file 12877_2023_3770_MOESM2_ESM.docx]

**Characterising complex health needs and the use of preventive therapies in the older population: a population-based cohort analysis of UK primary care and hospital linked data**

**Additional File 2**

[Table S1 descriptive statistics of variables used to identify cohorts 2](#_Toc123818261)

[Figure S1 Histograms of variables used to identify cohorts of complex health needs: (a) unplanned hospital admission, (b) eFI and (c) polypharmacy 2](#_Toc123818262)

[Figure S2 Prevalence of Charlson morbidity index deficits/conditions - in the year prior to start - for all cohorts, overlap and background population 3](#_Toc123818263)

[Table S2a: Point prevalence of the different antihypertensives classes for the Hospitalisation cohort 4](#_Toc123818264)

[Table S2b: Point prevalence of the different antihypertensives classes for the frailty cohort 5](#_Toc123818265)

[Table S2c: Point prevalence of the different antihypertensives classes for the polypharmacy cohort 6](#_Toc123818266)

[Table S2d: Point prevalence of the different antihypertensives classes for the overlap group 7](#_Toc123818267)

[Table S2e: Point prevalence of the different antihypertensives classes for the background population 8](#_Toc123818268)

[Table S3a: One-year IRs – excluding one-year prevalence of the different antihypertensives classes for the Hospitalisation cohort 9](#_Toc123818269)

[Table S3b: One-year IRs – excluding one-year prevalence of the different antihypertensives classes for the frailty cohort 10](#_Toc123818270)

[Table S3c: One-year IRs – excluding one-year prevalence of the different antihypertensives classes for the polypharmacy cohort 11](#_Toc123818271)

[Table S3d: One-year IRs – excluding one-year prevalence of the different antihypertensives classes for the overlap group 12](#_Toc123818272)

[Table S3e: One-year IRs – excluding one-year prevalence of the different antihypertensives classes for the background population 13](#_Toc123818273)

Table S1 and Figure S1 describe the distribution of the healthcare markers used to identify the cohorts to create the cohorts.

Table S1 descriptive statistics of variables used to identify cohorts

| **Overall population (n=475371)** | | | | | | |
| --- | --- | --- | --- | --- | --- | --- |
| **Variable** | **Min** | **P20** | **P40** | **P60** | **P80** | **Max** |
| **Unplanned hospital admission^1a^** | 0 | 0 | 0 | 0 | 1 | 76 |
| **eFI^2b^** | 0 | 1 | 2 | 3 | 4 | 14 |
| **Polypharmacy^3^** | 0 | 3 | 6 | 8 | 11 | 52 |

1 Defined by the number of unplanned hospital admissions (through accidents and emergency rooms or admitted patient care), identified in the linked HES data in 2009

^a^ 384 774 (80.9%) of the overall population did not have recorded unplanned hospital admissions in 2009

2 Defined using the validated eFI score developed by Clegg et al(5). and currently used by the NHS to support routine frailty identification(8). The eFI was calculated based on a count of frailty markers/deficits as recorded during 2009 in CPRD using a pre-specified list of Read codes(5).

^b^ 50 126 (10.5%) of the overall population had an eFI score ≥4 in 2009. A total of 110 225 (23.2%) had an eFI score ≥3 in 2009, hence, 3 was the cut-off chosen to identify the frailty cohort

3 Defined by the number of different drug substances prescribed in 2009. For each patient, all prescriptions issued by GPs in 2009 were identified and for these prescriptions, the drug substance was retrieved using the “PRODUCT” dictionary and “THERAPY” table in CPRD. Fixed combinations of multiple substances in products were counted as one substance.

P20, P40, P60, and P80: 20^th^, 40^th^, 60^th^, and 80^th^ percentile. eFI = electronic Frailty Index


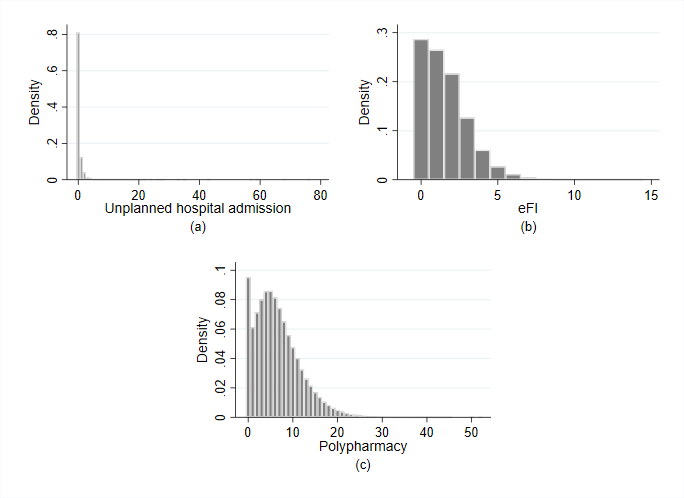


Figure S1 Histograms of variables used to identify cohorts of complex health needs: (a) unplanned hospital admission, (b) eFI and (c) polypharmacy


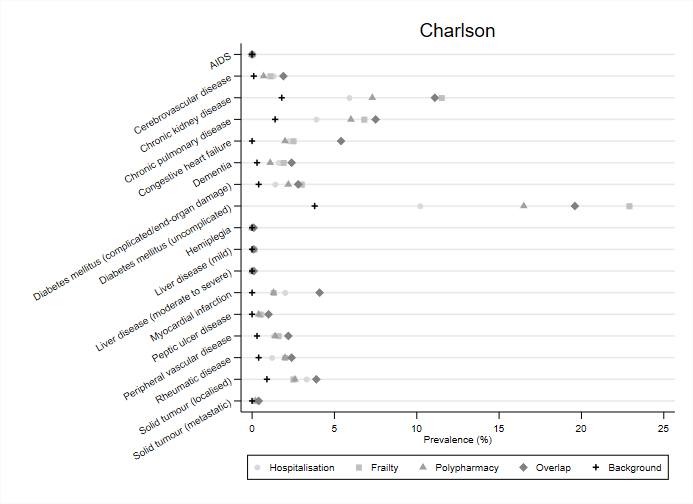


Figure S2 Prevalence of Charlson morbidity index deficits/conditions - in the year prior to start - for all cohorts, overlap and background population


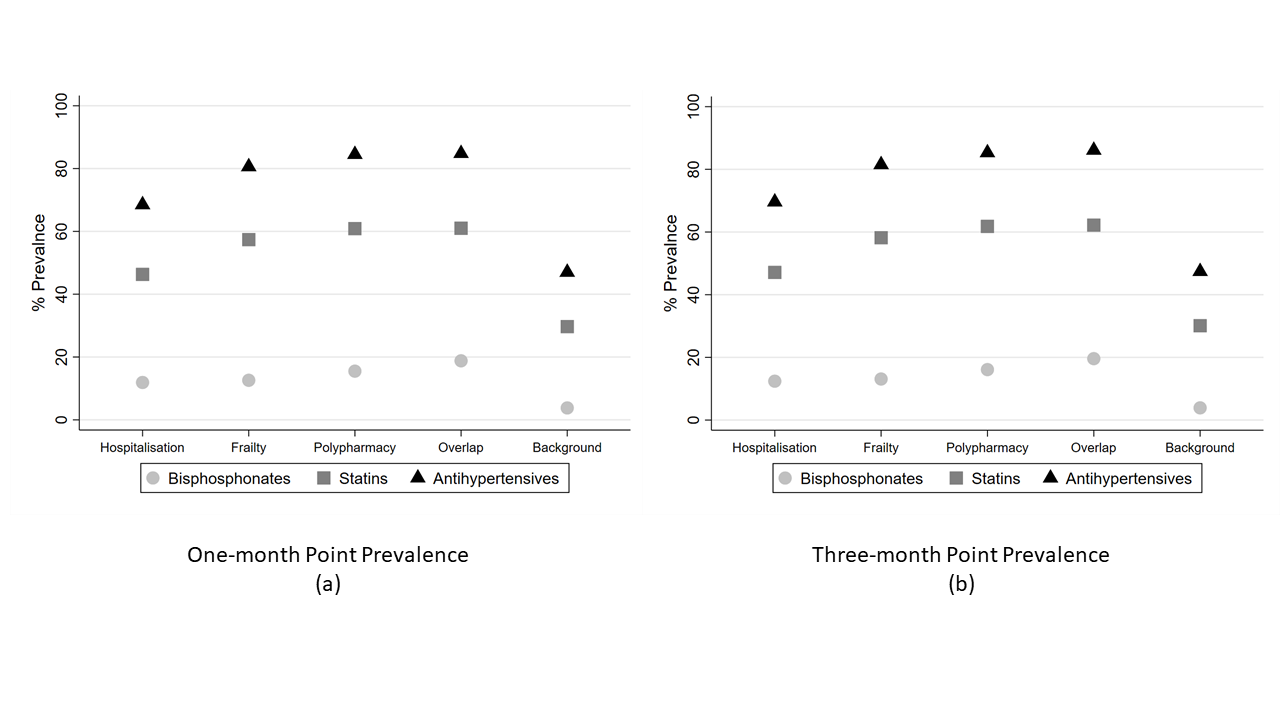
Figure S3 Point Prevalence for oral bisphosphonates, statins and anti-hypertensives, for all cohorts at (a) 1 month and (b) 3 months

Table S2a: Point prevalence of the different antihypertensives classes for the Hospitalisation cohort

| **Hospitalisation cohort N=90597** | | | | | | |
| --- | --- | --- | --- | --- | --- | --- |
| **Drug name** | **1 month PP** | | **3 months PP** | | **1 year PP** | |
|  | **N prevalent** | **PP (95% CI)** | **N prevalent** | **PP (95% CI)** | **N prevalent** | **PP (95% CI)** |
| ACE-I + CCB | 11 | 0 (0, 0) | 11 | 0 (0, 0) | 15 | 0 (0, 0) |
| ACE-I + Diuretics | 281 | 0.3 (0.3, 0.3) | 300 | 0.3 (0.3, 0.4) | 388 | 0.4 (0.4, 0.5) |
| ARB + CCB | 24 | 0 (0, 0) | 25 | 0 (0, 0) | 26 | 0 (0, 0) |
| ARB + Diuretics | 141 | 0.2 (0.1, 0.2) | 149 | 0.2 (0.1, 0.2) | 180 | 0.2 (0.2, 0.2) |
| Betablockers + CCB | 26 | 0 (0, 0) | 27 | 0 (0, 0) | 32 | 0 (0, 0) |
| Betablockers + Diuretics | 303 | 0.3 (0.3, 0.4) | 323 | 0.4 (0.3, 0.4) | 422 | 0.5 (0.4, 0.5) |
| Renin-Inhibitors | 60 | 0.1 (0.1, 0.1) | 63 | 0.1 (0.1, 0.1) | 87 | 0.1 (0.1, 0.1) |
| ACE-I | 27588 | 30.5 (30.1, 30.8) | 28486 | 31.4 (31.1, 31.8) | 31419 | 34.7 (34.3, 35.1) |
| ARB | 11724 | 12.9 (12.7, 13.2) | 12045 | 13.3 (13.1, 13.5) | 13076 | 14.4 (14.2, 14.7) |
| All antihypertensives | 62056 | 68.5 (68.0, 69.0) | 63017 | 69.6 (69.0, 70.1) | 65387 | 72.2 (71.6, 72.7) |
| Other antihypertensives* | 5427 | 6.0 (5.8, 6.2) | 5749 | 6.3 (6.2, 6.5) | 6760 | 7.5 (7.3, 7.6) |
| Betablockers | 22774 | 25.1 (24.8, 25.5) | 23406 | 25.8 (25.5, 26.2) | 25467 | 28.1 (27.8, 28.5) |
| CCB | 23376 | 25.8 (25.5, 26.1) | 24378 | 26.9 (26.6, 27.2) | 27540 | 30.4 (30.0, 30.8) |
| Diuretics | 32672 | 36.1 (35.7, 36.5) | 34173 | 37.7 (37.3, 38.1) | 38126 | 42.1 (41.7, 42.5) |

Note: ACE-I = Angiotensin-converting-enzyme inhibitor, CCB = calcium channel blockers, ARB = Angiotensin-II receptor blockers. *other antihypertensives comprise: Ambrisentan, Betanidine, Bosentan, Clonidine,Debrisoquine, Doxazosin,Guanethidine, Hydralazine, Indoramin, Ketanserin, Macitentan, Methoserpidine ,Methyldopa, Methyldopate, Metirosine, Minoxidil, Moxonidine, Prazosin, Reserpine, Riociguat, Sitaxentan, Sodium nitroprusside and Trimetaphan camsilate

Table S2b: Point prevalence of the different antihypertensives classes for the frailty cohort

| **Frailty cohort N=110225** | | | | | | |
| --- | --- | --- | --- | --- | --- | --- |
| **Drug name** | **1 month PP** | | **3 months PP** | | **1 year PP** | |
|  | **N prevalent** | **PP (95% CI)** | **N prevalent** | **PP (95% CI)** | **N prevalent** | **PP (95% CI)** |
| ACE-I + CCB | 29 | 0 (0, 0) | 30 | 0 (0, 0) | 38 | 0 (0, 0) |
| ACE-I + Diuretics | 473 | 0.4 (0.4, 0.5) | 501 | 0.5 (0.4, 0.5) | 617 | 0.6 (0.5, 0.6) |
| ARB + CCB | 22 | 0 (0, 0) | 23 | 0 (0, 0) | 23 | 0 (0, 0) |
| ARB + Diuretics | 228 | 0.2 (0.2, 0.2) | 238 | 0.2 (0.2, 0.2) | 301 | 0.3 (0.2, 0.3) |
| Betablockers + CCB | 30 | 0 (0, 0) | 31 | 0 (0, 0) | 40 | 0 (0, 0) |
| Betablockers + Diuretics | 484 | 0.4 (0.4, 0.5) | 501 | 0.5 (0.4, 0.5) | 623 | 0.6 (0.5, 0.6) |
| Renin-Inhibitors | 123 | 0.1 (0.1, 0.1) | 131 | 0.1 (0.1, 0.1) | 154 | 0.1 (0.1, 0.2) |
| ACE-I | 42538 | 38.6 (38.2, 39.0) | 43651 | 39.6 (39.2, 40.0) | 47382 | 43.0 (42.6, 43.4) |
| ARB | 19638 | 17.8 (17.6, 18.1) | 20027 | 18.2 (17.9, 18.4) | 21325 | 19.3 (19.1, 19.6) |
| All antihypertensives | 88863 | 80.6 (80.1, 81.2) | 89797 | 81.5 (80.9, 82.0) | 92014 | 83.5 (82.9, 84.0) |
| Other antihypertensives* | 9739 | 8.8 (8.7, 9.0) | 10137 | 9.2 (9.0, 9.4) | 11480 | 10.4 (10.2, 10.6) |
| Betablockers | 31101 | 28.2 (27.9, 28.5) | 31767 | 28.8 (28.5, 29.1) | 34106 | 30.9 (30.6, 31.3) |
| CCB | 35338 | 32.1 (31.7, 32.4) | 36522 | 33.1 (32.8, 33.5) | 40453 | 36.7 (36.3, 37.1) |
| Diuretics | 48364 | 43.9 (43.5, 44.3) | 50160 | 45.5 (45.1, 45.9) | 55022 | 49.9 (49.5, 50.3) |

Note: ACE-I = Angiotensin-converting-enzyme inhibitor, CCB = calcium channel blockers, ARB = Angiotensin-II receptor blockers. *other antihypertensives comprise: Ambrisentan, Betanidine, Bosentan, Clonidine,Debrisoquine, Doxazosin,Guanethidine, Hydralazine, Indoramin, Ketanserin, Macitentan, Methoserpidine ,Methyldopa, Methyldopate, Metirosine, Minoxidil, Moxonidine, Prazosin, Reserpine, Riociguat, Sitaxentan, Sodium nitroprusside and Trimetaphan camsilate

Table S2c: Point prevalence of the different antihypertensives classes for the polypharmacy cohort

| **Polypharmacy cohort N=116076** | | | | | | |
| --- | --- | --- | --- | --- | --- | --- |
| **Drug name** | **1 month PP** | | **3 months PP** | | **1 year PP** | |
|  | **N prevalent** | **PP (95% CI)** | **N prevalent** | **PP (95% CI)** | **N prevalent** | **PP (95% CI)** |
| ACE-I + CCB | 18 | 0 (0, 0) | 19 | 0 (0, 0) | 33 | 0 (0, 0) |
| ACE-I + Diuretics | 503 | 0.4 (0.4, 0.5) | 536 | 0.5 (0.4, 0.5) | 660 | 0.6 (0.5, 0.6) |
| ARB + CCB | 33 | 0 (0, 0) | 34 | 0 (0, 0) | 36 | 0 (0, 0) |
| ARB + Diuretics | 308 | 0.3 (0.2, 0.3) | 326 | 0.3 (0.3, 0.3) | 396 | 0.3 (0.3, 0.4) |
| Betablockers + CCB | 29 | 0 (0, 0) | 32 | 0 (0, 0) | 39 | 0 (0, 0) |
| Betablockers + Diuretics | 461 | 0.4 (0.4, 0.4) | 490 | 0.4 (0.4, 0.5) | 620 | 0.5 (0.5, 0.6) |
| Renin-Inhibitors | 173 | 0.1 (0.1, 0.2) | 183 | 0.2 (0.1, 0.2) | 215 | 0.2 (0.2, 0.2) |
| ACE-I | 45732 | 39.4 (39.0, 39.8) | 46947 | 40.4 (40.1, 40.8) | 50964 | 43.9 (43.5, 44.3) |
| ARB | 23327 | 20.1 (19.8, 20.4) | 23785 | 20.5 (20.2, 20.8) | 25216 | 21.7 (21.5, 22.0) |
| All antihypertensives | 98069 | 84.5 (84.0, 85.0) | 98995 | 85.3 (84.8, 85.8) | 101121 | 87.1 (86.6, 87.7) |
| Other antihypertensives* | 11867 | 10.2 (10.0, 10.4) | 12373 | 10.7 (10.5, 10.8) | 13995 | 12.1 (11.9, 12.3) |
| Betablockers | 37388 | 32.2 (31.9, 32.5) | 38131 | 32.9 (32.5, 33.2) | 40731 | 35.1 (34.7, 35.4) |
| CCB | 41720 | 35.9 (35.6, 36.3) | 43092 | 37.1 (36.8, 37.5) | 47568 | 41.0 (40.6, 41.3) |
| Diuretics | 57259 | 49.3 (48.9, 49.7) | 59339 | 51.1 (50.7, 51.5) | 64681 | 55.7 (55.3, 56.2) |

Note: ACE-I = Angiotensin-converting-enzyme inhibitor, CCB = calcium channel blockers, ARB = Angiotensin-II receptor blockers. *other antihypertensives comprise: Ambrisentan, Betanidine, Bosentan, Clonidine,Debrisoquine, Doxazosin,Guanethidine, Hydralazine, Indoramin, Ketanserin, Macitentan, Methoserpidine ,Methyldopa, Methyldopate, Metirosine, Minoxidil, Moxonidine, Prazosin, Reserpine, Riociguat, Sitaxentan, Sodium nitroprusside and Trimetaphan camsilate

Table S2d: Point prevalence of the different antihypertensives classes for the overlap group

| **Overlap group N=28259** | | | | | | |
| --- | --- | --- | --- | --- | --- | --- |
| **Drug name** | **1 month PP** | | **3 months PP** | | **1 year PP** | |
|  | **N prevalent** | **PP (95% CI)** | **N prevalent** | **PP (95% CI)** | **N prevalent** | **PP (95% CI)** |
| ACE-I + CCB | 6 | 0 (0, 0) | 6 | 0 (0, 0) | 8 | 0 (0, 0.001) |
| ACE-I + Diuretics | 94 | 0.3 (0.3, 0.4) | 106 | 0.4 (0.3, 0.5) | 145 | 0.5 (0.4, 0.6) |
| ARB + CCB | 6 | 0 (0, 0) | 7 | 0 (0, 0.1) | 7 | 0 (0, 0.1) |
| ARB + Diuretics | 41 | 0.1 (0.1, 0.2) | 45 | 0.2 (0.1, 0.2) | 61 | 0.2 (0.2, 0.3) |
| Betablockers + CCB | 0 | NA | <5 | NA | <5 | NA |
| Betablockers + Diuretics | 51 | 0.2 (0.1, 0.2) | 57 | 0.2 (0.2, 0.3) | 103 | 0.4 (0.3, 0.4) |
| Renin-Inhibitors | 31 | 0.1 (0.1, 0.2) | 31 | 0.1 (0.1, 0.2) | 43 | 0.2 (0.1, 0.2) |
| ACE-I | 11314 | 40.0 (39.3, 40.8) | 11818 | 41.8 (41.1, 42.6) | 13253 | 46.9 (46.1, 47.7) |
| ARB | 5254 | 18.6 (18.1, 19.1) | 5435 | 19.2 (18.7, 19.8) | 6016 | 21.3 (20.8, 21.8) |
| All antihypertensives | 23957 | 84.8 (83.7, 85.9) | 24323 | 86.1 (85.0, 87.2) | 25112 | 88.9 (87.8, 90.0) |
| Other antihypertensives* | 2559 | 9.1 (8.7, 9.4) | 2741 | 97. (9.3, 10.1) | 3300 | 11.7 (11.3, 12.1) |
| Betablockers | 9314 | 33.0 (32.3, 33.6) | 9626 | 34.1 (33.4, 34.8) | 10562 | 37.4 (36.7, 38.1) |
| CCB | 9208 | 32.6 (31.9, 33.3) | 9737 | 34.5 (33.8, 35.1) | 11318 | 40.1 (39.3, 40.8) |
| Diuretics | 14835 | 52.5 (51.7, 53.3) | 15547 | 55.0 (54.2, 55.9) | 17300 | 61.2 (60.3, 62.1) |

Note: ACE-I = Angiotensin-converting-enzyme inhibitor, CCB = calcium channel blockers, ARB = Angiotensin-II receptor blockers. *other antihypertensives comprise: Ambrisentan, Betanidine, Bosentan, Clonidine,Debrisoquine, Doxazosin,Guanethidine, Hydralazine, Indoramin, Ketanserin, Macitentan, Methoserpidine ,Methyldopa, Methyldopate, Metirosine, Minoxidil, Moxonidine, Prazosin, Reserpine, Riociguat, Sitaxentan, Sodium nitroprusside and Trimetaphan camsilate

Table S2e: Point prevalence of the different antihypertensives classes for the background population

| **Background population N=277332** | | | | | | |
| --- | --- | --- | --- | --- | --- | --- |
| **Drug name** | **1 month PP** | | **3 months PP** | | **1 year PP** | |
|  | **N prevalent** | **PP (95% CI)** | **N prevalent** | **PP (95% CI)** | **N prevalent** | **PP (95% CI)** |
| ACE-I + CCB | 50 | 0 (0, 0) | 52 | 0 (0, 0) | 57 | 0 (0, 0) |
| ACE-I + Diuretics | 1052 | 0.4 (0.4, 0.4) | 1066 | 0.4 (0.4, 0.4) | 1155 | 0.4 (0.4, 0.4) |
| ARB + CCB | 25 | 0 (0, 0) | 27 | 0 (0, 0) | 30 | 0 (0, 0) |
| ARB + Diuretics | 426 | 0.2 (0.1, 0.2) | 436 | 0.2 (0.1, 0.2) | 459 | 0.2 (0.2, 0.2) |
| Betablockers + CCB | 120 | 0 (0, 0.1) | 121 | 0 (0, 0.1) | 124 | 0 (0, 0.1) |
| Betablockers + Diuretics | 1634 | 0.6 (0.6, 0.6) | 1663 | 0.6 (0.6, 0.6) | 1798 | 0.6 (0.6, 0.7) |
| Renin-Inhibitors | 83 | 0 (0, 0) | 89 | 0 (0, 0) | 99 | 0 (0, 0) |
| ACE-I | 54058 | 19.5 (19.3, 19.7) | 54653 | 19.7 (19.5, 19.9) | 57730 | 20.8 (20.6, 21.0) |
| ARB | 21582 | 7.8 (7.7, 7.9) | 21762 | 7.8 (7.7, 8.0) | 22541 | 8.1 (8.0, 8.2) |
| All antihypertensives | 130411 | 47.0 (46.8, 47.3) | 131354 | 47.4 (47.1, 47.6) | 134675 | 48.6 (48.3, 48.8) |
| Other antihypertensives* | 9762 | 3.5 (3.5, 3.6) | 9959 | 3.6 (3.5, 3.7) | 10781 | 3.9 (3.8, 4.0) |
| Betablockers | 41161 | 14.8 (14.7, 15.0) | 41629 | 15.0 (14.9, 15.2) | 43793 | 15.8 (15.6, 15.9) |
| CCB | 51332 | 18.5 (18.3, 18.7) | 52055 | 18.8 (18.6, 18.9) | 55234 | 19.9 (19.8, 20.1) |
| Diuretics | 56577 | 20.4 (20.2, 20.6) | 57695 | 20.8 (20.6, 21.0) | 61844 | 22.3 (22.1, 22.5) |

Note: ACE-I = Angiotensin-converting-enzyme inhibitor, CCB = calcium channel blockers, ARB = Angiotensin-II receptor blockers. *other antihypertensives comprise: Ambrisentan, Betanidine, Bosentan, Clonidine,Debrisoquine, Doxazosin,Guanethidine, Hydralazine, Indoramin, Ketanserin, Macitentan, Methoserpidine ,Methyldopa, Methyldopate, Metirosine, Minoxidil, Moxonidine, Prazosin, Reserpine, Riociguat, Sitaxentan, Sodium nitroprusside and Trimetaphan camsilate

Table S3a: One-year IRs – excluding one-year prevalence of the different antihypertensives classes for the Hospitalisation cohort

| **Drug name** | **Hospitalisation cohort N=90597** | | |
| --- | --- | --- | --- |
|  | **N incidence** | **PY** | **IR (95% CI)** |
| **ACE-I + CCB** | <5 | NA | NA |
| **ACE-I + Diuretics** | 18 | 81946.7 | 0.2 (0.1, 0.3) |
| **ARB + CCB** | 9 | 82284.4 | 0.1 (0.0, 0.2) |
| **ARB + Diuretics** | 13 | 82145.1 | 0.2 (0.1, 0.2) |
| **Betablockers + CCB** | 0 | NA | NA |
| **Betablockers + Diuretics** | <5 | NA | NA |
| **Renin-Inhibitors** | 25 | 82217.3 | 0.3 (0.2, 0.4) |
| **ACE-I** | 2905 | 52211.7 | 55.6 (53.6, 57.7) |
| **ARB** | 1230 | 69601.9 | 17.7 (16.7, 18.7) |
| **All antihypertensives** | 2793 | 21610.1 | 129.3 (124.5, 134.0) |
| **Other antihypertensives*** | 848 | 75686.8 | 11.2 (10.5, 12.0) |
| **Betablockers** | 2445 | 57871.6 | 42.3 (40.6, 43.9) |
| **CCB** | 2783 | 55607.2 | 50.1 (48.2, 51.9) |
| **Diuretics** | 4141 | 46198.4 | 89.6 (86.9, 92.4) |

PY=person years

Note: ACE-I = Angiotensin-converting-enzyme inhibitor, CCB = calcium channel blockers, ARB = Angiotensin-II receptor blockers. *other antihypertensives comprise: Ambrisentan, Betanidine, Bosentan, Clonidine,Debrisoquine, Doxazosin,Guanethidine, Hydralazine, Indoramin, Ketanserin, Macitentan, Methoserpidine ,Methyldopa, Methyldopate, Metirosine, Minoxidil, Moxonidine, Prazosin, Reserpine, Riociguat, Sitaxentan, Sodium nitroprusside and Trimetaphan camsilate

Table S3b: One-year IRs – excluding one-year prevalence of the different antihypertensives classes for the frailty cohort

| **Drug name** | **Frailty cohort N=110225** | | |
| --- | --- | --- | --- |
|  | **N incidence** | **PY** | **IR (95% CI)** |
| **ACE-I + CCB** | 5 | 101713.0 | 0.1 (0.0, 0.1) |
| **ACE-I + Diuretics** | 29 | 101161.0 | 0.3 (0.2, 0.4) |
| **ARB + CCB** | 19 | 101717.8 | 0.2 (0.1, 0.3) |
| **ARB + Diuretics** | 32 | 101448.5 | 0.3 (0.2, 0.3) |
| **Betablockers + CCB** | <5 | NA | NA |
| **Betablockers + Diuretics** | 8 | 101159.6 | 0.1 (0.0, 0.1) |
| **Renin-Inhibitors** | 43 | 101577.2 | 0.4 (0.3, 0.6) |
| **ACE-I** | 3400 | 55998.0 | 60.7 (58.7, 62.8) |
| **ARB** | 1716 | 80840.6 | 21.2 (20.2, 22.2) |
| **All antihypertensives** | 2400 | 15449.0 | 155.4 (149.1, 161.6) |
| **Other antihypertensives*** | 1291 | 90359.1 | 14.3 (13.5, 15.1) |
| **Betablockers** | 2933 | 68671.5 | 42.7 (41.2, 44.3) |
| **CCB** | 3334 | 62256.8 | 53.6 (51.7, 55.4) |
| **Diuretics** | 5102 | 48913.8 | 104.3 (101.4, 107.2) |

PY=person years

Note: ACE-I = Angiotensin-converting-enzyme inhibitor, CCB = calcium channel blockers, ARB = Angiotensin-II receptor blockers. *other antihypertensives comprise: Ambrisentan, Betanidine, Bosentan, Clonidine,Debrisoquine, Doxazosin,Guanethidine, Hydralazine, Indoramin, Ketanserin, Macitentan, Methoserpidine ,Methyldopa, Methyldopate, Metirosine, Minoxidil, Moxonidine, Prazosin, Reserpine, Riociguat, Sitaxentan, Sodium nitroprusside and Trimetaphan camsilate

Table S3c: One-year IRs – excluding one-year prevalence of the different antihypertensives classes for the polypharmacy cohort

| **Drug name** | **Polypharmacy cohort N=116076** | | |
| --- | --- | --- | --- |
|  | **N incidence** | **PY** | **IR (95% CI)** |
| **ACE-I + CCB** | 5 | 107295.7 | 0.05 (0.01, 0.09) |
| **ACE-I + Diuretics** | 40 | 106684.8 | 0.38 (0.26, 0.49) |
| **ARB + CCB** | 16 | 107286.8 | 0.15 (0.08, 0.22) |
| **ARB + Diuretics** | 39 | 106932.9 | 0.37 (0.25, 0.48) |
| **Betablockers + CCB** | <5 | NA | NA |
| **Betablockers + Diuretics** | 12 | 106732.5 | 0.11 (0.05, 0.18) |
| **Renin-Inhibitors** | 53 | 107091.2 | 0.50 (0.36, 0.63) |
| **ACE-I** | 3339 | 58366.1 | 57.21 (55.27, 59.15) |
| **ARB** | 1936 | 82641.2 | 23.43 (22.38, 24.47) |
| **All antihypertensives** | 1972 | 12759.6 | 154.55 (147.73, 161.37) |
| **Other antihypertensives*** | 1454 | 93430.1 | 15.56 (14.76, 16.36) |
| **Betablockers** | 2894 | 68095.8 | 42.50 (40.95, 44.05) |
| **CCB** | 3368 | 61092.4 | 55.13 (53.27, 56.99) |
| **Diuretics** | 4983 | 45487.4 | 109.55 (106.51, 112.59) |

PY=person years

Note: ACE-I = Angiotensin-converting-enzyme inhibitor, CCB = calcium channel blockers, ARB = Angiotensin-II receptor blockers. *other antihypertensives comprise: Ambrisentan, Betanidine, Bosentan, Clonidine,Debrisoquine, Doxazosin,Guanethidine, Hydralazine, Indoramin, Ketanserin, Macitentan, Methoserpidine ,Methyldopa, Methyldopate, Metirosine, Minoxidil, Moxonidine, Prazosin, Reserpine, Riociguat, Sitaxentan, Sodium nitroprusside and Trimetaphan camsilate

Table S3d: One-year IRs – excluding one-year prevalence of the different antihypertensives classes for the overlap group

| **Drug name** | **Overlap group N=28259** | | |
| --- | --- | --- | --- |
|  | **N incidence** | **PY** | **IR (95% CI)** |
| **ACE-I + CCB** | 0 | NA | NA |
| **ACE-I + Diuretics** | 5 | 24729.1 | 0.20 (0.03, 0.38) |
| **ARB + CCB** | <5 | NA | NA |
| **ARB + Diuretics** | 7 | 24802.3 | 0.28 (0.07, 0.49) |
| **Betablockers + CCB** | 0 | NA | NA |
| **Betablockers + Diuretics** | <5 | NA | NA |
| **Renin-Inhibitors** | 9 | 24814.1 | 0.36 (0.13, 0.6) |
| **ACE-I** | 866 | 12665.3 | 68.38 (63.82, 72.9) |
| **ARB** | 472 | 19174.3 | 24.62 (22.40, 26.84) |
| **All antihypertensives** | 491 | 2481.9 | 197.83 (180.33, 215.33) |
| **Other antihypertensives*** | 315 | 21729.2 | 14.50 (12.90, 16.10) |
| **Betablockers** | 782 | 15105.2 | 51.77 (48.14, 55.40) |
| **CCB** | 828 | 14269.9 | 58.03 (54.08, 61.98) |
| **Diuretics** | 1381 | 9117.8 | 151.46 (143.47, 159.45) |

PY=person years

Note: ACE-I = Angiotensin-converting-enzyme inhibitor, CCB = calcium channel blockers, ARB = Angiotensin-II receptor blockers. *other antihypertensives comprise: Ambrisentan, Betanidine, Bosentan, Clonidine,Debrisoquine, Doxazosin,Guanethidine, Hydralazine, Indoramin, Ketanserin, Macitentan, Methoserpidine ,Methyldopa, Methyldopate, Metirosine, Minoxidil, Moxonidine, Prazosin, Reserpine, Riociguat, Sitaxentan, Sodium nitroprusside and Trimetaphan camsilate

Table S3e: One-year IRs – excluding one-year prevalence of the different antihypertensives classes for the background population

| **Drug name** | **Background population N=277332** | | |
| --- | --- | --- | --- |
|  | **N incidence** | **PY** | **IR (95% CI)** |
| **ACE-I + CCB** | 5 | 265656.0 | 0.02 (0, 0.04) |
| **ACE-I + Diuretics** | 79 | 264560.6 | 0.30 (0.23, 0.36) |
| **ARB + CCB** | 25 | 265671.2 | 0.09 (0.06, 0.13) |
| **ARB + Diuretics** | 33 | 265244.8 | 0.12 (0.08, 0.17) |
| **Betablockers + CCB** | <5 | NA | NA |
| **Betablockers + Diuretics** | 24 | 263954.9 | 0.09 (0.06, 0.13) |
| **Renin-Inhibitors** | 45 | 265589.5 | 0.17 (0.12, 0.22) |
| **ACE-I** | 7348 | 206580.2 | 35.57 (34.76, 36.38) |
| **ARB** | 2400 | 242782.7 | 9.89 (9.49, 10.28) |
| **All antihypertensives** | 9622 | 131709.3 | 73.06 (71.60, 74.52) |
| **Other antihypertensives*** | 1553 | 254571.4 | 6.10 (5.80, 6.40) |
| **Betablockers** | 5137 | 221275.9 | 23.22 (22.58, 23.85) |
| **CCB** | 7555 | 208727.9 | 36.20 (35.38, 37.01) |
| **Diuretics** | 8059 | 202584.7 | 39.78 (38.91, 40.65) |

PY=person years

Note: ACE-I = Angiotensin-converting-enzyme inhibitor, CCB = calcium channel blockers, ARB = Angiotensin-II receptor blockers. *other antihypertensives comprise: Ambrisentan, Betanidine, Bosentan, Clonidine,Debrisoquine, Doxazosin,Guanethidine, Hydralazine, Indoramin, Ketanserin, Macitentan, Methoserpidine ,Methyldopa, Methyldopate, Metirosine, Minoxidil, Moxonidine, Prazosin, Reserpine, Riociguat, Sitaxentan, Sodium nitroprusside and Trimetaphan camsilate
